# Fight smarter, not harder: NPR1-mediated immune balance in citrus greening disease

**DOI:** 10.1093/plphys/kiaf479

**Published:** 2025-10-07

**Authors:** Ritu Singh, Marcella Teixeira

**Affiliations:** Assistant Features Editor, Plant Physiology, American Society of Plant Biologists; Department of Plant Science, University of California, Davis, CA 95616, USA; Assistant Features Editor, Plant Physiology, American Society of Plant Biologists; Department of Plant Pathology, Washington State University, Pullman, WA 99163, USA

Huanglongbing (HLB), or citrus greening, is a devastating disease caused by the bacterium *Candidatus* Liberibacter asiaticus (*C*Las). The pathogen is spread by psyllids, sap-sucking insects that feed from citrus plants. A major challenge in combating HLB is that the citrus plant's own immune system contributes to the disease's severity. Infected plants overreact to the pathogen, triggering reactive oxygen species (ROS) outburst and excessive callose deposition, a carbohydrate that plugs phloem pores. This uncontrolled defense response, rather than stopping the pathogen, leads to collapse of the phloem, obstructing the transport of photoassimilates (Ma et al. 2022; Welker and Levy 2022), resulting in stunted growth and yellowing leaves (Schneider 1968; da Graça et al. 2016).

Recent studies suggest that the key to fighting HLB lies, not in a stronger immune response, but in a smarter one. Comparisons between susceptible and tolerant citrus varieties suggest that systemic acquired resistance, a salicylic acid (SA)–dependent systemic immune response, is key to HLB tolerance (Wang et al. 2016; Welker and Levy 2022). SA is central to systemic acquired resistance signaling and is perceived by NONEXPRESSOR OF PATHOGENESIS-RELATED GENES 1, 3, and 4 (NPR1, NPR3, and NPR4). While NPR1 activates the defense-related genes, NPR3 and 4 play opposite roles to modulate this activity, highlighting the importance of a finely tuned balanced immune response (Ding et al. 2018; Kumar et al. 2022). Furthermore, NPR1-like genes are induced in the tolerant variety but not the susceptible (Martinelli et al. 2012). Interestingly, genetically engineering susceptible citrus with the *NPR1* gene from the model plant *Arabidopsis* (*AtNPR1*) successfully confers tolerance to the disease (Dutt et al. 2015). This leads to a central paradox: how can boosting a master immune activator (*NPR1*) fix a disease caused by an overactive immune system? Understanding this mechanism is key to developing a sustainable defense against citrus greening.

In this issue of *Plant Physiology*, Sarkar and colleagues (2025) uncovered the mechanism behind this paradox. They first pinpointed the timing of immune activation in citrus plants during *C*Las infection. By infecting sweet orange (Hamlin) and grapefruit (Duncan) using psyllids, they observed callose deposition within just 1 d of infection, while ROS accumulation was observed at 14 d postinoculation. This established 14 d postinoculation as a critical window to study citrus immune responses.

The authors then investigated how the immune regulator *AtNPR1* mediates tolerance to HLB. To address this question, the authors evaluated callose and ROS accumulation after inoculating wild type Hamlin and Duncan trees, as well as trees engineered to overexpress Arabidopsis *NPR1* (*AtNPR1-OE*), with either *C*Las-free or *C*Las-infected psyllids. In wild type citrus, *C*Las infection triggered massive callose and ROS accumulation, which ultimately clogged phloem and exacerbated disease. In contrast, *AtNPR1-OE* plants behaved differently: without infection, these plants maintained slightly elevated basal callose levels, but infection no longer triggered uncontrolled callose or ROS accumulation. As a result, after infection, ROS and callose levels remained far lower in the engineered trees than in wild type, preserving phloem function.

Microscopic examination of vascular anatomy confirmed these differences. Infected wild type plants showed substantial expansion of phloem and xylem tissues and extensive callose deposition that sealed sieve pores, whereas *AtNPR1-OE* plants exhibited only moderate vascular changes and retained visible pore openings. Furthermore, biochemical analysis revealed that wild type plants accumulated high levels of SA upon infection, while *AtNPR1-OE* plants showed only a mild increase.

To confirm this *AtNPR1*-mediated immune regulation, the team pursued a second strategy. They silenced *CsNPR3*, a citrus gene that represses SA signaling. Silencing *CsNPR3* phenocopied *AtNPR1* overexpression: higher basal callose deposition suppressed infection-triggered callose and ROS accumulation and reduced HLB symptom development. This suggests that *CsNPR3* plays a positive role in HLB disease development.

Finally, to determine whether this *NPR1*-mediated immune balance is conserved in other pathosystems, the group extended its investigation to *Arabidopsis thaliana* challenged with *Pseudomonas syringae* (*Psm*). They compared how wild type plants, *AtNPR1-OE*, and *npr1–3* mutants responded to *Psm-*induced callose and ROS accumulation. As in citrus, *AtNPR1* overexpression increased basal callose levels but suppressed pathogen-triggered callose and ROS, while *npr1* mutants showed uncontrolled ROS accumulation. Moreover, infection of the double mutant *npr3/npr4* with *Psm* resulted in similar outcomes, demonstrating that NPR proteins fine-tune the balance between basal and induced defenses.

Together, these findings identify *NPR1* as a central brake that prevents citrus immunity from spiraling out of control (Figure). By maintaining steady basal callose levels while curbing excessive ROS and SA accumulation, *NPR1* enables plants to tolerate *C*Las infection without self-destruction. This dual role, activating and restraining, underscores the importance of immune homeostasis in long-term survival. The authors propose that HLB susceptibility arises from immune imbalance in vulnerable citrus varieties, which could be corrected by *NPR1* overexpression or *NPR3* repression. Mechanistically, *NPR1* likely maintains immune homeostasis through feedback inhibition of SA biosynthesis, thereby preventing excessive induction of callose and ROS biosynthesis-related genes (Lee et al. 2020).

**Figure.**
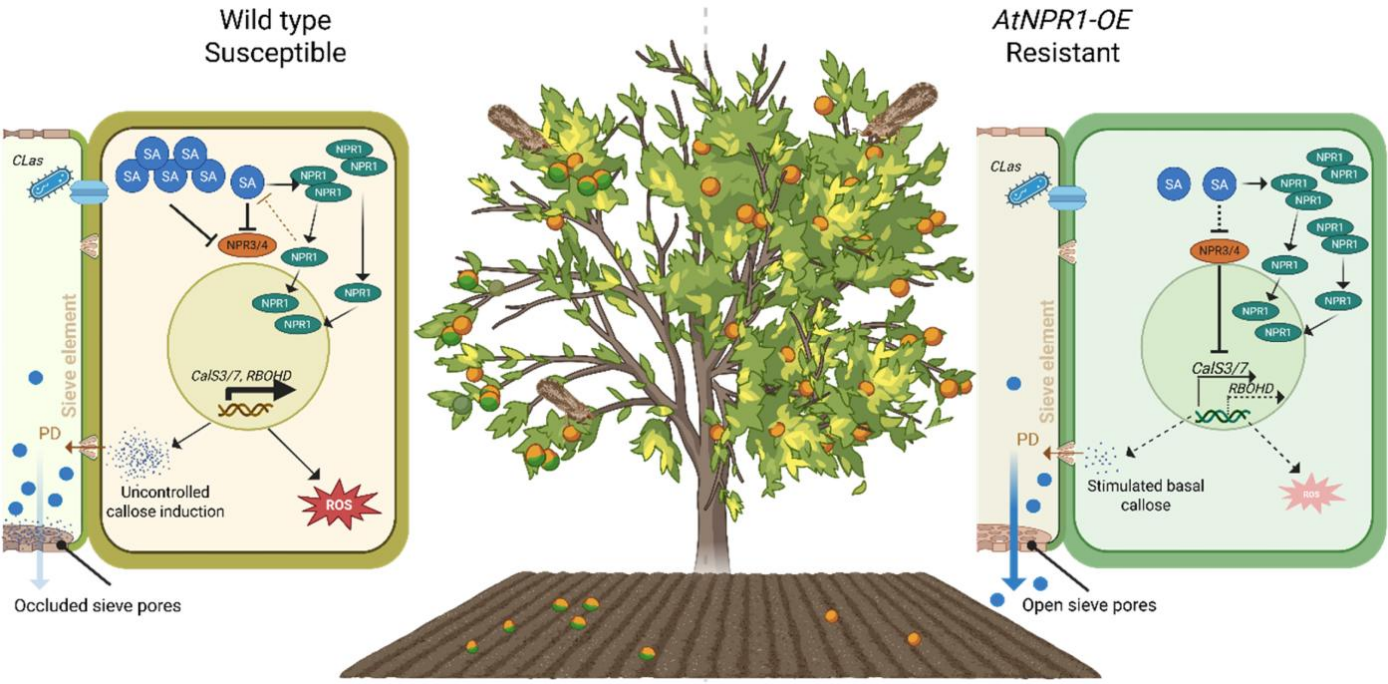
Wild type response and NPR overexpression-mediated tolerance to HLB in citrus. Susceptible citrus plants show an overreaction to *C*Las infection, which results in uncontrolled callose accumulation and ROS burst (left side, wild type tree). These events lead to sieve element occlusion and reduced nutrient flow (light arrow on the left side sieve element). When citrus plants overexpressing *AtNPR1* are infected with *C*Las, the high level of NPR1 results in suppression of SA accumulation, inhibiting callose deposition (via expression of callose synthase genes *CalS3/7*) and ROS accumulation (via expression of *RBOHD*), which ultimately prevents complete sieve plate occlusion (dark arrow on the right side sieve element). The balanced immunity allows proper nutrient flow and tolerance to *C*Las infection. Adapted from Figure 8 of Sarkar et al. (2025). Created in BioRender. https://BioRender.com/87ugwhm.

## Data Availability

No data are generated in this study.
